# Effect of Pig-Adipose-Derived Stem Cells’ Conditioned Media on Skin Wound-Healing Characteristics In Vitro

**DOI:** 10.3390/ijms22115469

**Published:** 2021-05-22

**Authors:** Joanna Wiśniewska, Magda Słyszewska, Karolina Stałanowska, Katarzyna Walendzik, Marta Kopcewicz, Sylwia Machcińska, Barbara Gawrońska-Kozak

**Affiliations:** 1 Department of Biological Function of Food, Institute of Animal Reproduction and Food Research, Polish Academy of Sciences, 10-748 Olsztyn, Poland; m.slyszewska@pan.olsztyn.pl (M.S.); k.walendzik@pan.olsztyn.pl (K.W.); m.kopcewicz@pan.olsztyn.pl (M.K.); s.machcinska@pan.olsztyn.pl (S.M.); b.kozak@pan.olsztyn.pl (B.G.-K.); 2 Department of Plant Physiology, Genetics, and Biotechnology, Faculty of Biology and Biotechnology, University of Warmia and Mazury, 10-719 Olsztyn, Poland; karolina.stalanowska@uwm.edu.pl

**Keywords:** adipose-derived stem cells, skin cells, wound healing, hypoxia, secretome, cell therapy

## Abstract

The primary mechanism by which adipose-derived stem cells (ASCs) exert their reparative or regenerative potential relies predominantly on paracrine action. Secretory abilities of ASCs have been found to be amplified by hypoxia pre-conditioning. This study investigates the impact of hypoxia (1% O_2_) on the secretome composition of pig ASCs (pASCs) and explores the effect of pASCs’ conditioned media (CM) on skin cell functions in vitro and the expression of markers attributed to wound healing. Exposure of pASCs to hypoxia increased levels of vascular endothelial growth factor (VEGF) in CM-Hyp compared to CM collected from the cells cultured in normoxia (CM-Nor). CM-Hyp promoted the migratory ability of pig keratinocytes (pKERs) and delayed migration of pig dermal fibroblasts (pDFs). Exposure of pKERs to either CM-Nor or CM-Hyp decreased the levels of pro-fibrotic indicators *WNT10A* and *WNT11*. Furthermore, CM-Hyp enhanced the expression of *KRT14*, the marker of the basal epidermis layer. In contrast, CM-Nor showed a stronger effect on pDFs manifested by increases in *TGFB1*, *COL1A1*, *COL3A1*, and *FN1* mRNA expression. The formation of three-dimensional endothelial cell networks was improved in the presence of CM-Hyp. Overall, our results demonstrate that the paracrine activity of pASCs affects skin cells, and this property might be used to modulate wound healing.

## 1. Introduction

The recent past has brought considerable advances in technologies to improve wound healing (e.g., skin grafting, negative pressure wound therapy (NPWT), hyperbaric oxygen therapy (HBO)) that significantly reduce the burden of a variety of cutaneous wounds. Although these modalities have notably improved the outcome of healing, to date there are no efficient paths with the potential to overcome the formation of pathologic or regular scars at the site of injury (tissue repair) and the ability to convert the repair into scar-free healing (regeneration). In mammals, including humans, the rare phenomenon of skin regeneration occurs in fetuses [1] and is exhibited by a very limited number of adult representatives, such as Foxn1-deficient (nude) mice [2,3,4] and African spiny mice (*Acomys*) [5]. Existing studies demonstrate that regeneration of skin wounds in nude mice is primarily associated with the defect in the expression of the transcription factor Foxn1 in the suprabasal layer of the epidermis [4,6]. Key indicators of post-wounded skin regeneration are evidenced by, among other things, higher ratio of collagen type III (Col3α1) to type I (Col1α1) [7], high levels of fibronectin (Fn) and tenascin C (Tnc) [5], reduced expression of transforming growth factor beta 1 (Tgfβ1), and an increase in Tgfβ3 content [8]. On the contrary, regular cutaneous wound repair involves excessive deposition of extracellular matrix (ECM), mainly collagen type I [9,10], an increase in Tgfβ1 level [8], and differentiation of dermal fibroblasts (DFs) into myofibroblasts [11]. Additionally, recent studies on mouse models of wound healing demonstrate that the Wnt pathway contributes to the cellular response to the injury and promotes the development of scar tissue [12,13]. We have previously shown that the expression levels of Wnt10a and Wnt11, the representatives of Wnt/β-catenin-dependent and Wnt/β-catenin-independent pathways, respectively, increase early during the remodeling phase and might contribute to ECM deposition during cutaneous healing in mice [13].

Accumulating data involving animal models [14] and human clinical cases [15,16] indicate that adipose-derived stem cell (ASC) therapy provides a promising approach in wound healing improvement. It has been demonstrated that delivery of ASCs at the site of skin injury augments healing by enhancing re-epithelialization, angiogenesis, and dermal repair [14,17]. Although ASCs show the ability to differentiate into functional cells that restore skin integrity [18], the main mechanism by which the cells mediate an effect on the wound relies predominantly on the paracrine activities of the cells [19]. Several soluble factors, including vascular endothelial growth factor (VEGF), basic fibroblast growth factor (bFGF), insulin-like growth factor-1 (IGF-1), hepatocyte growth factor (HGF), tumor necrosis growth factor-α (TNF-α), and interleukins (ILs)-6, -7, -8, and -11, have been identified in conditioned media (CM) collected from human and rodent cultures of ASC [20,21,22,23]. Moreover, the secretory activity of ASCs exerts an anti-apoptotic effect [24] and regulates skin cell function in vitro [25]. Recent studies by Kim et al. [26] show that CM collected from 2D- and 3D-ASC cultures increases the proliferation and accelerates the migration of human dermal fibroblasts (HDF) and HaCaT cells. Another report provides evidence that ASC-CM promotes the formation of capillary-like structures by human dermal lymphatic endothelial cells (HDLECs) [20].

A large body of data reveals that exposing ASCs to low oxygen (hypoxia) amplifies their paracrine activity, particularly with respect to angiogenesis enhancement [27,28]. In addition, it has been demonstrated that the delivery of hypoxic ASC-CM to cutaneous wounds in mice reduces their size and depth relative to wounds treated with standard, normoxic ASC-CM [28]. Our previous studies on pig ASCs (pASCs) demonstrate that hypoxia (1% O_2_) alters the expression of proteins associated with cell metabolism, ECM components, and intracellular communication [29]. Moreover, functional examination of pASCs pre-conditioned with hypoxia exhibits the acquisition of contractile abilities in vitro. These hypoxia-caused changes might enhance the potential of pASCs to improve wound healing. However, the effect of the pASCs secretome has not been examined in terms of skin cell functions in vitro.

Here, we investigated the effect of hypoxia (1% O_2_) on a secretome of ASCs isolated from the subcutaneous fat of domestic pigs (pASCs). Next, we demonstrated the impact of the two CM forms of pASCs (i.e., the CM obtained from pASCs cultured under normoxia (CM-Nor) or hypoxia (CM-Hyp) conditions) on the functional characteristics of skin cellular components, including porcine keratinocytes (pKERs), dermal fibroblast (pDFs), and human umbilical vein endothelial cells (HUVEC). Furthermore, we examined the effect of pASC CM forms on the expression of cutaneous wound-healing indicators, including members of Wnt and Tgfβ pathways, as well as specific epidermal and dermal markers that reflect pro-regenerative or pro-reparative properties of skin cells. Together, our study indicates that the pASC secretome, containing VEGF, bFGF, and MCP-1, affects skin cells and may provide a promising strategy to promote wound healing.

## 2. Results

### 2.1. Characterization of Protein Secreted by pASCs

**Figure 1:**
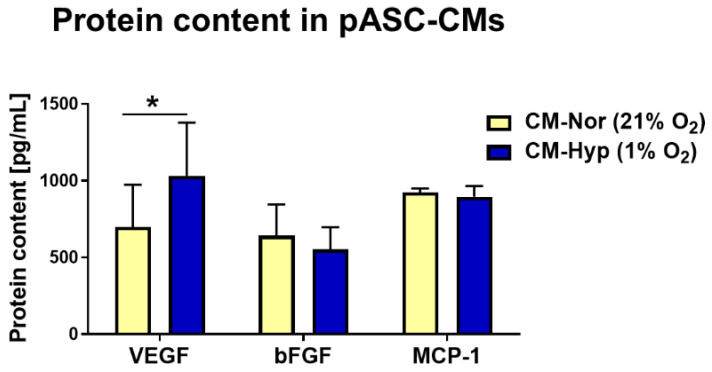
Bead-based quantification of protein levels secreted by pASCs cultured at 24 h in normoxia (21% O_2_; yellow bars; *n* = 7) or hypoxia (1% O_2_; blue bars; *n* = 7). Data are expressed as mean ± SD. * *p* < 0.05. Abbreviations: pASCs, pig adipose-derived stem cell; CM-Nor, conditioned medium collected from pASCs exposed to normoxia; CM-Hyp, conditioned medium collected from pASCs cultured under hypoxia.

To determine the effect of hypoxia (1% O_2_) or normoxia (21% O_2_) on pASCs paracrine activity with respect to proteins associated with wound healing, both types of pASC-CMs (CM-Nor, CM-Hyp) were analyzed based on the CBA method. In total, 12 proteins were measured (VEGF, bFGF, IL-1α, IL-1β, IL-2, IL-4, IL-5, IL-9, IL-10, MCP-1, RANTES, and GM-CSF). Hypoxia significantly increased VEGF secretion into CM when compared to normoxia-exposed pASCs (*p* < 0.05; Figure 1). There were no differences in bFGF and MCP-1 content between CM-Nor and CM-Hyp (Figure 1). The remaining proteins were not observed at detectable levels. These results indicate that VEGF, bFGF, and MCP-1 comprise the major components of the pASC secretome, and of these, VEGF levels were elevated upon hypoxia.

### 2.2. Effect of pASC-CM on pKERs and pDFs Migration and Contractility

**Figure 2:**
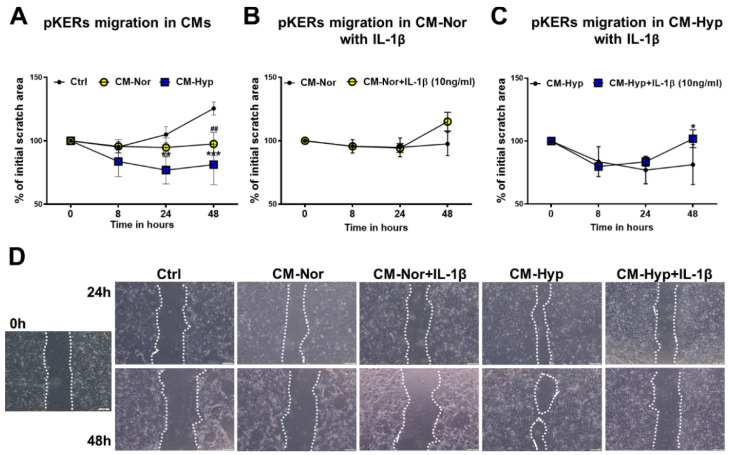
Effect of pASCs-CMs alone or supplemented with IL-1β (10 ng/mL) on migratory abilities of pKERs. Wound-healing scratch assay demonstrates increased cell migration upon administration with CM-Hyp (**A**). Supplementation of CM-Nor (**B**) or CM-Hyp (**C**) with IL-1β did not further increase pKERs motility relative to the cultures exposed to CMs alone. (**D**) Representative images depicting the stimulatory effect of CM-Hyp on pKERs movement at different time points (0, 24 and 48 h), and lack of effect of both CMs upon supplementation with IL-1β. The results are shown as the mean ± SD. The asterisks indicate significant differences between CM-Hyp exposed cells and control cultures (**A**) or CM-Hyp vs CM-Hyp+ IL-1β (**C**) (* *p* < 0.05; ** *p* < 0.01; *** *p* < 0.001). Hashes show significant differences between pKERs cultured in CM-Nor and control medium (^##^ *p* < 0.01). Scale bars = 100 μm (**D**). Abbreviations: pASCs, pig adipose-derived stem cell; pKERs, pig keratinocytes; CM-Nor, conditioned medium collected from pASCs exposed to normoxia; CM-Hyp, conditioned medium collected from pASCs cultured under hypoxia; IL-1β, interleukin 1 beta.

**Figure 3:**
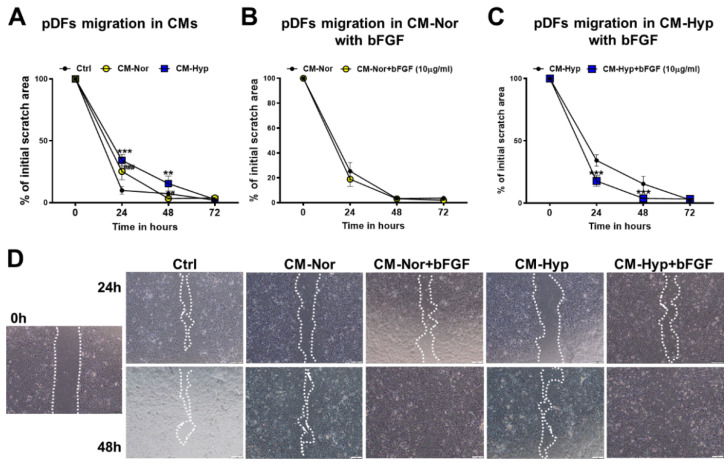
Effect of pASCs-CMs alone or supplemented with bFGF (10 µg/mL) on pDFs migration. Wound healing scratch assay shows decreased migration of pDFs exposed to both CM-Nor and CM-Hyp (**A**). Supplementation of (**B**) CM-Nor with bFGF has no effect on cells’ motility; however, (**C**) bFGF enrichment of CM-Hyp improved cells’ motility at 24 and 48 h. (**D**) Representative pictures demonstrating the stimulatory effect of CMs and bFGF on pDFs’ migratory abilities at different time points (0, 24 and 48 h). The results are shown as the mean ± SD. The asterisks indicate significant differences between CM-Hyp exposed pDFs and control cultures (**A**) or CM-Hyp vs CM-Hyp+ bFGF (**C**) (** *p* < 0.01; *** *p* < 0.001). Hashes show significant differences between pDFs cultured in CM-Nor and the control medium (^#^ *p* < 0.05; ^###^ *p* < 0.001). Abbreviations: pASCs, pig adipose-derived stem cell; pDFs, pig dermal fibroblasts; CM-Nor, conditioned medium collected from pASCs exposed to normoxia; CM-Hyp, conditioned medium collected from pASCs cultured under hypoxia; bFGF, basic fibroblasts growth factor.

Cells’ migratory capabilities are essential for proper wound closure and re-establishment of the new tissue at the site of injury. The impact of pASC-CMs on migratory properties of pKERs (Figure 2) and pDFs (Figure 3) was examined with a scratch assay. Initially, pKERs were exposed for 48 h to CM-Hyp, CM-Nor, or the control medium. The administration of CM-Hyp led to a gradual increase in cell migration over a time course relative to the effect observed at the onset of the experiment, which was designated as time 0 (Figure 2A,D; Supplementary Table S2). Moreover, the administration of CM-Hyp significantly increased the cell migration at 24 and 48 h compared to control cultures (*p* < 0.01 and *p* < 0.001 for 24 and 48 h, respectively; Figure 2A,D). No changes with time were found in migratory abilities of pKERs exposed to CM-Nor (Figure 2A,D; Supplementary Table S2). However, pKERs stimulated with control medium (Figure 2A,D) and to a lesser extent those exposed to CMs showed a gradual detachment from the growth surface that prevented extended monitoring (longer than 48 h) of the cell migration. Therefore, CMs were supplemented with IL-1β (10 ng/mL) because this pro-inflammatory cytokine acts as an initiator of keratinocyte activation, stimulating them to migrate and hyperproliferate [30,31]. Neither CM-Nor nor CM-Hyp enriched with IL-1β improved the ability of pKERs to migrate when compared with the effect exerted by non-supplemented CMs (Figure 2B,C, respectively). Moreover, the presence of IL-1β in both CMs increased the rate of the cells’ detachment at 48 h of culture relative to consecutive days of the experiment (*p* < 0.05 for CM-Hyp supplemented with IL-1β; Figure 2B–D; Supplementary Tables S3 and S4).

The pDFs’ cultures showed continuous closure of the wound (scratch assay) over time that was completed at 72 h regardless of the type of media (Figure 3A,D; Supplementary Table S5). However, pDFs exposed to CM-Hyp exhibited a delay in gap closure at 24 h (*p* < 0.001; Figure 3A,D) and 48 h (*p* < 0.01; Figure 3A,D) compared to control cultures. It is well established that bFGF promotes fibroblast migration in the multiple wound healing models in vitro [32,33]. Therefore, next, we supplemented CMs with bFGF (10 µg/mL), even though the bead-based analysis revealed detectable levels of bFGF expression in both types of CMs (see Figure 1). As depicted in Figure 3B–D, supplementation of both CM-Nor and CM-Hyp with bFGF maintained the ability of pDFs to close the wound gradually with time (Supplementary Tables S6 and S7 for CM-Nor or CM-Hyp, respectively). However, bFGF enrichment of CM-Hyp improved cell motility at 24 and 48 h compared with CM-Hyp alone (*p* < 0.001 for both 24 and 48 h; Figure 3C,D). Together, these results demonstrate that CM-Hyp had a greater effect than CM-Nor on skin cell migration. In addition, differences between pKERs’ and pDFs’ migration upon CM-Hyp treatment suggest a cell-specific response to the pASC secretome.

**Figure 4:**
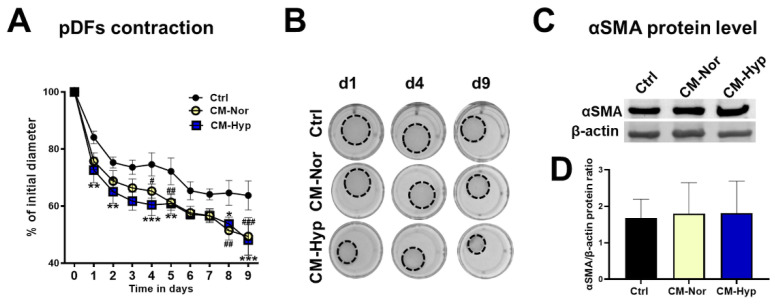
Contractile abilities of pDFs exposed to pASC-CMs. (**A**) Both CM-Nor and CM-Hyp increased pDFs capacity to contract collagen lattices as compared to control cultures. (**B**) Representative images show pDFs contractile abilities in CM-Nor, CM-Hyp, and control medium at days 1, 4, and 9. (**C**) Western blot and (**D**) densitometry analysis of total αSMA protein content detected in DFs exposed to CMs. The results are shown as the mean ± SD. The asterisks indicate significant differences between CM-Hyp treated and control cultures (* *p* < 0.05; ** *p* < 0.01; *** *p* < 0.001). Hashes show significant differences between CM-Nor exposed cells and control (^#^ *p* < 0.05; ^##^ *p* < 0.01; ^###^ *p* < 0.001). Abbreviations: pASCs, pig adipose-derived stem cell; pDFs, pig dermal fibroblasts; CM-Nor, conditioned medium collected from pASCs exposed to normoxia; CM-Hyp, conditioned medium collected from pASCs cultured under hypoxia; αSMA, smooth muscle actin alpha.

Next, the contractile abilities of pDFs that are essential for wound closure in vivo were evaluated using a gel contraction assay. The size of collagen lattices showed progressive reduction over time regardless of the type of media (Figure 4A,B; Supplementary Table S8). However, culture with either CM-Nor or CM-Hyp caused a significant decrease in gel matrices’ size compared to control media (Figure 4A,B). The gel contraction rate showed similarities between pDFs exposed to CM-Nor or CM-Hyp, with statistically significant increases detected for CM-Hyp at days 1, 2, 4, 5, 8, and 9 (*p* < 0.05; Figure 4A,B) and for CM-Nor at days 4, 5, 8, and 9 (*p* < 0.05; Figure 4A,B). At day 9, collagen lattices reduced their initial size to 48.09 ± 10.76% and 49.38 ± 13.12% upon treatment with CM-Hyp or CM-Nor, respectively. In contrast, control gels contracted to 63.73 ± 10.15% of their original area (Figure 4A,B). However, Western blot analysis showed no effect on pro-contractile αSMA protein expression in pDFs cultured for 48 h in both CM-Nor or CM-Hyp compared to control medium-treated cells (Figure 4C,D). In conclusion, both CM forms improved pDFs contractile abilities, suggesting they may be useful in promoting wound closure via contraction in vivo.

### 2.3. Effect of pASC-CMs on the mRNA Expression of Wnt Pathway Components and Keratins in pKERs

To assess whether pASC-CMs impact genes associated with wound repair and those ones that are involved in keratinocytes differentiation and turnover, we investigated the mRNA expression profiles of the Wnt pathway ligands *WNT10A*, *WNT11*, and the Wnt direct target gene *AXIN2*, and the panel of keratins attributed specifically to the basal (*KRT14*), differentiating (*KRT1*, *KRT10*), differentiated (*IVL*), and activated (*KRT6A, KRT17*) keratinocytes.

**Figure 5:**
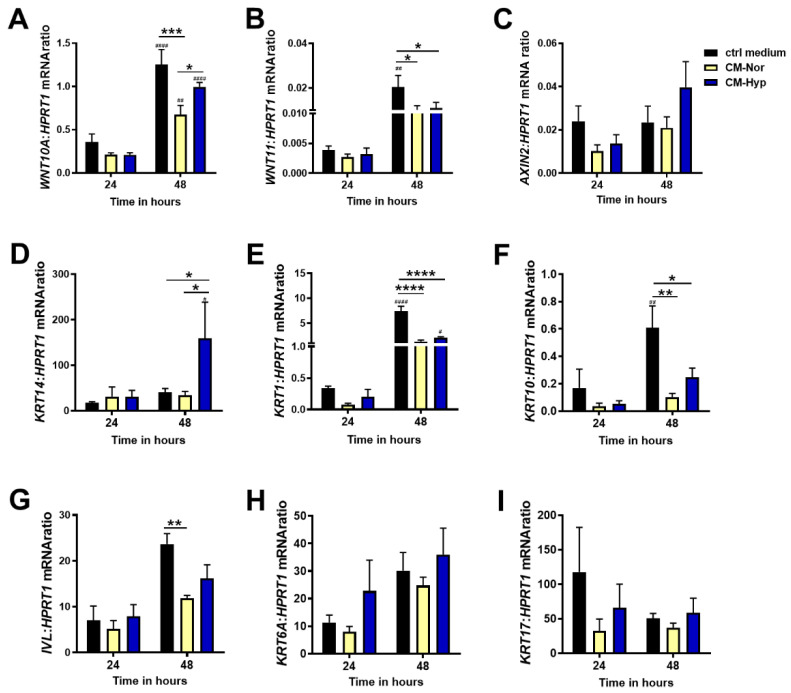
Quantitative analysis of (**A**) *WNT10A*, (**B**) *WNT11*, (**C**) *AXIN2*, (**D**) *KRT14*, (**E**) *KRT1*, (**F**) *KRT10*, (**G**) *IVL*, (**H**) *KRT6A*, and (**I**) *KRT17* mRNA expression in pKERs exposed for 24 and 48 h to CM-Nor (yellow bars) or CM-Hyp (blue bars) relative to control-treated cells (black bars). The results are shown as the mean ± SD. The asterisks indicate significant differences between specific mRNA expression at 48 h (* *p* < 0.05; ** *p* < 0.01; *** *p* < 0.001; **** *p* < 0.0001). Hashes show significant differences in mRNA between 24 and 48 h of culture (^#^ *p* < 0.05, ^##^ *p* < 0.01, ^####^ *p* < 0.0001). Abbreviations: pKERs, pig keratinocytes; CM-Nor, conditioned medium collected from pASCs exposed to normoxia; CM-Hyp, conditioned medium collected from pASCs cultured under hypoxia.

Generally, the significant changes in mRNA expression for all examined transcripts were observed at 48 h rather than 24 h, indicating long-term responses of pKERs to pASC-CMs administration (Figure 5). Exposure to both CM-Nor or CM-Hyp decreased the levels of pro-fibrotic indicators, *WNT10A* (*p* < 0.001 for CM-Nor; Figure 5A) and *WNT11* (*p* < 0.05 for CM-Nor and CM-Hyp, Figure 5B), in comparison to the control medium, whereas no effect on the expression of the Wnt target gene *AXIN2* was detected (Figure 5C). The presence of CM-Hyp significantly increased the mRNA levels of *KRT14*, the marker of the basal keratinocytes layer, when compared to the control- and CM-Nor-treated cultures (*p* < 0.05; Figure 5D). On the contrary, both CMs reduced the levels of *KRT1* (*p* < 0.0001; Figure 5E), *KRT10* (*p* < 0.01; *p* < 0.05 for CM-Nor and CM-Hyp, respectively; Figure 5F), and *IVL* (*p* < 0.01 for CM-Nor; Figure 5G) relative to the control pKERs. CMs had no effect on the mRNA levels of activated keratins including *KRT6A* (Figure 5H) and *KRT17* (Figure 5I). Thus, both CM forms may affect pKER turnover, leading to a decrease in cell differentiation, while CM-Hyp exclusively supported self-renewal.

### 2.4. Effect of pASC-CMs on the mRNA Expression of Wnt and Tgfβ Pathway Members, ECM Components, and CCN2 in pDFs Cultures

**Figure 6:**
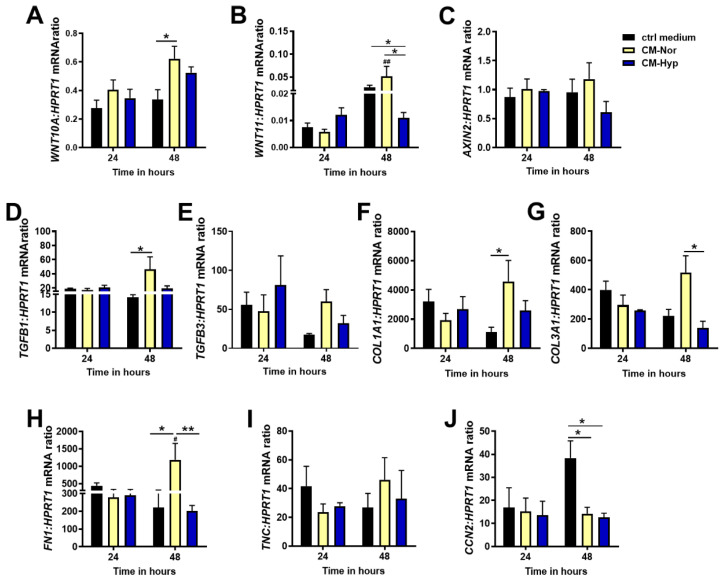
Quantitative analysis of: (**A**) *WNT10A*, (**B**) *WNT11*, (**C**) *AXIN2*, (**D**) *TGFB1*, (**E**) *TGFB3*, (**F**) *COL1A1*, (**G**) *COL3A1*, (**H**) *FN1*, (**I**) *TNC*, and (**J**) *CCN2* mRNA expression in pDFs exposed for 24 and 48 h to CM-Nor (yellow bars) or CM-Hyp (blue bars) relative to control-treated cells (black bars). The results are shown as the mean ± SD. The asterisks indicate significant differences between specific mRNA expression at 48 h (* *p* < 0.05; ** *p* < 0.01). Hashes show significant differences in mRNA between 24 and 48 h of culture (^#^ *p* < 0.05; ^##^ *p* < 0.01). Abbreviations: pDFs, pig dermal fibroblasts; CM-Nor, conditioned medium collected from pASCs exposed to normoxia; CM-Hyp, conditioned medium collected from pASCs cultured under hypoxia.

To gain further insight into the cellular response of the dermal compartment to pASC-CMs exposure, we evaluated the mRNA levels of molecules that comprise pathways contributing to the outcome of cutaneous healing (scar-ended reparation or scarless regeneration) that are represented by *WNT10A*, *WNT11*, *AXIN2*, *TGFB1*, and *TGFB3*. Moreover, we examined the expression of ECM protein transcripts that are involved in skin restoration, including *COL1A1*, *COL3A1*, *FN1*, and *TNC*, by forming a scar at the site of injury. We also determined the levels of connective tissue growth factors (*CCN2*; *CTGF*) that impact scar tissue formation [34] (Figure 6).

Similar to pKERs, significant changes in the pDFs response to the presence of CMs were detected at 48 h but not after 24 h of treatment. Administration of pDFs with both CMs markedly increased *WNT10A* mRNA compared to control cultures (*p* < 0.05 for CM-Nor; Figure 6A). A contrasting effect of CMs was observed for *WNT11* expression. Whereas CM-Nor upregulated expression of the *WNT11* transcript, exposure to CM-Hyp led to its suppression (*p* < 0.05; Figure 6B). Neither CM-Nor nor CM-Hyp had an effect on *AXIN2* mRNA levels (Figure 6C). Furthermore, CM-Nor but not CM-Hyp significantly elevated the mRNA levels of pro-fibrotic marker *TGFB1* (*p* < 0.05; Figure 6D). Similarly, pro-regenerative *TGFB3* mRNA showed a tendency to increase upon CM-Nor treatment (Figure 6E).

With regard to the expression of ECM representatives, our study revealed that CM-Nor upregulated mRNA of *COL1A1* relative to the control group (*p* < 0.05; Figure 6F) and its presence increased expression of pro-regenerative *COL3A1* compared to the effect caused by CM-Hyp (*p* < 0.05; Figure 6G). Similarly, the transcripts representing other ECM components including *FN1* (*p* < 0.05; Figure 6H) and *TNC* (non-significant; Figure 6I) were susceptible exclusively to CM-Nor administration. In contrast, exposure to both CM-Hyp or CM-Nor significantly decreased the level of *CCN2* mRNA, which produces a protein that is a mediator and marker of tissue fibrosis (*p* < 0.05; Figure 6J). These data indicate that CM-Nor, but not CM-Hyp, promotes the expression of pro-scarring mediators (Wnts) and ECM components stimulating regular (scar-forming) wound healing.

### 2.5. Effect of pASC-CMs on Endothelial Cells Function

**Figure 7:**
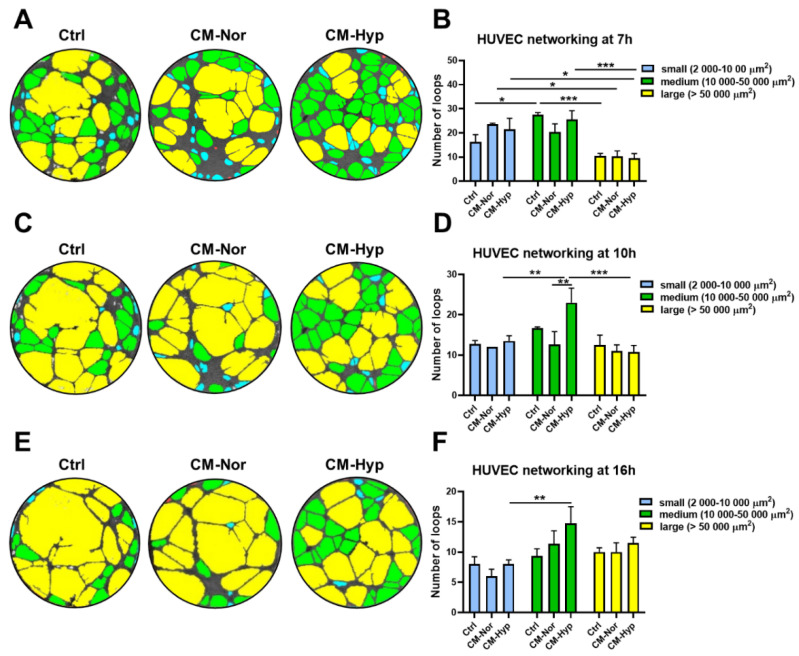
CM-Hyp increases formation and survival of HUVEC networking. Representative pictures of endothelial cells tube formation in CM-Nor, CM-Hyp, or control medium and their analysis at (**A**,**B**) 7 h, (**C**,**D**) 10 h, and (**E**,**F**) 16 h of culture demonstrated small-(blue), medium-(green), and large- (yellow) sized loops. The results are shown as the mean ± SD. The asterisks indicate significant differences in loops number between specific media (* *p* < 0.05, ** *p* < 0.01, *** *p* < 0.001). Abbreviations: HUVEC, human umbilical vein endothelial cells; CM-Nor, conditioned medium collected from pASCs exposed to normoxia; CM-Hyp, conditioned medium collected from pASCs cultured under hypoxia.

Because bead-based analysis of CMs revealed the presence of VEGF (see Figure 1), particularly in CM-Hyp, we next investigated the capacity of CMs to reinforce angiogenesis in vitro. The tube formation assay of HUVEC revealed the presence of three groups of endothelial loops, (i.e., small (2000–10,000 µm^2^), medium (10,000–50,000 µm^2^), and large (>50,000 µm^2^), regardless of the type of medium (Figure 7). However, differences in the loops’ distribution between cultures treated with CM-Nor, CM-Hyp, and control medium were observed. Medium-sized tubes represented the most abundant type of endothelial network regardless of the 7-, 10-, or 16-h period (Figure 7A–F). At 10 h after cell seeding, the number of medium-sized loops significantly increased upon stimulation with CM-Hyp compared to CM-Nor exposure cultures (*p* < 0.01; Figure 7C,D) and relative to the amount of small (*p* < 0.01; Figure 7C,D) or large (*p* < 0.001; Figure 7C,D) loops developed in CM-Hyp. Similarly, at a longer time lapse (16 h), CM-Hyp continued to maintain the high number of medium-sized tubes compared to CM-Nor and control-treated cells (not significant) and relative to the small loops (*p* < 0.01; Figure 7E,F). Thus, CM-Hyp had a greater than CM-Nor pro-angiogenic activity by extending the maintenance of medium-sized cell loops during the experimental time course.

## 3. Discussion

Advances in stem cell research show that therapeutic effects implemented by ASCs are primarily attributed to their trophic action on the secretion of a vast range of soluble factors [20,21,23]. Subsequently, conditioning of ASCs by exposing them to hypoxia has been frequently used to enhance the paracrine function of human ASCs (hASCs) [27,28]. Our previous report demonstrates the impact of oxygen deficiency on pASCs proteome and their functionality in vitro [29]. The present study focused on the effect of hypoxia (1% O_2_) on pASCs secretome and CMs bioactivity in terms of their potential to modulate skin cells’ characteristics, particularly those that are associated with wound healing.

Extensive previous results have provided evidence that ASCs secrete a panel of cytokines and growth factors, among which VEGF represents the most abundantly expressed protein [27,28,35]. Similar to results with hASCs, our study demonstrates that hypoxia significantly elevated VEGF production by pASCs (see Figure 1). This paracrine production of VEGF, which is an important pro-angiogenic and pro-survival factor, provides fundamental mechanisms for tissue repair initiated by cell-based or cell-free therapies, as has been shown in numerous models of injury in skin, heart, and brain [28,35,36,37]. To further investigate the functional improvement of human endothelial cells cultured in VEGF-rich pASC-CMs, we evaluated the formation of HUVEC tubular cell networks (see Figure 7). We found that CM-Hyp had a greater pro-angiogenic effect on HUVEC than CM-Nor by extending the maintenance of medium-sized cell loops during the time course of the experiment. However, pASCs exposed to normoxic culture conditions released basal levels of VEGF that also showed pro-angiogenic potential in vitro. These data suggest that production of VEGF constitutes innate characteristics of ASCs regardless of the cell donor species or oxygen availability and that the differences in VEGF levels between various pASCs-primed media lead to subtle, rather qualitative differences in their angiogenic activity. Furthermore, no differences in bFGF and MCP-1 (CCL2) content were found between CM-Nor and CM-Hyp (see Figure 1). Similar results for bFGF and MCP-1 were obtained in studies on hASCs and mouse bone-marrow mesenchymal stem cells (BM-MSCs), respectively [35,38]. Both factors are vital for successful wound healing. Indeed, the administration of bFGF to a rat model of cutaneous injury improved wound healing by stimulating fibroblast growth, and it regulated inflammatory response [39]. Others have reported that bFGF regulates the balance between collagen synthesis and degradation through the induction of matrix metalloproteinase 1 (MMP-1), and hence may ameliorate scar quality [40]. With regard to MCP-1, there is extensive literature suggesting its role as a potent chemoattractant for monocytes and macrophages during wound healing [41,42]. Proper macrophage activation and polarization are necessary for sufficient tissue repair [43]. An experimental study on C57BLKS-m *lepr^db^* (db/db) mice has shown that the impaired macrophage response in diabetic chronic wounds may result from chemokine insufficiency early after injury, whereas application of CCL2 increases immune cell infiltration and enhances wound healing [44]. In light of existing studies that underscore the great importance of factors secreted by human and mouse MSCs for wound-healing improvement, our findings are the first that have extended this concept to add pig ASCs to the list of stem cells with documented potency to produce therapeutic agents.

Cell migration is essential for cutaneous repair and constitutes a rate-limiting event during the wound healing process. We therefore investigated whether the pASCs’ secretome created by normoxic or hypoxic environments has the ability to enhance wound healing by activating the motility of skin cells (see Figure 2 and Figure 3). Although overall migration of pKERs was moderate, this capability was enhanced by CM-Hyp treatment (see Figure 2). Our results are consistent with the findings in other studies in which hASCs and BM-MSCs promote keratinocyte-based “wound healing” in an in vitro experimental system [38,45]. In order to mimic a wound environment that is characterized by high levels of pro-inflammatory cytokines, including IL-1β, that contribute to keratinocyte activation manifested by increased migration [46], we supplemented CMs with IL-1β for the assessment of pKERs migration. We found that IL-1β had no effect on pKER migration, but its presence increased the cells’ tendency toward detachment from the growth surface. One explanation that may account for this observation involves the specific requirements for an optimal cell culture of pKERs in terms of culture media composition and coating substrates. A recent study by Ponce et al., in which several culture media and adhesion substrates were examined, clearly demonstrates that media rich in hormones and specific growth factors together with the collagen-based matrix used to cover growth surface are necessary for the effective cultivation of keratinocytes isolated from the skin of newborn piglets [47]. In light of these findings, we cannot exclude that in our study, although CMs were rich in VEGF, bFGF, and MCP-1, they did not contain the full package of factors necessary for supporting pKERs’ migration. However, in a separate experiment, pKERs were exposed to the same CMs, and the cultures did not exhibit symptoms of detaching from the culture plate. This finding suggests that the unique cultivation requirements of pKERs are addressed exclusively to their migratory potential. Alternatively, blocking pKERs’ proliferation by using mitomycin C prior to the onset of the scratch assay may lead to partial inhibition of the cells’ migration and concomitantly force them to be detached from the plate surface.

In contrast to pKERs, the migration of pDFs was significantly suppressed by the presence of CM-Hyp and to a lesser extent by CM-Nor. Nevertheless, this inhibitory effect was reversed by CM-Hyp supplementation with an additional dose of bFGF (see Figure 3). Similar results have been shown in studies on rat and human primary DFs cultured in standard and high glucose conditions, respectively [32,48].

Wnt signaling plays a pivotal role in wound healing where it shows a strong association with scar formation and development of fibrosis [12,49]. Major recent studies indicate that the inhibition of Wnt/β-catenin signaling, either by using chemical blockers or by introducing adenoviral systems expressing soluble Wnt decoy receptor of LRP6 (sLRP6E1E2), leads to dermal fibrosis suppression in mouse models of cutaneous injury [12,50]. Accordingly, there is significant interest in finding means of modulation of Wnt signaling in damaged tissues that would provide a promising strategy for the treatment of pathologic scars like keloids or hypertrophic scars. In the current study, we demonstrated that mRNA expression of *WNT10A* and *WNT11* decreased in CM cultured pKERs, regardless of the CM type used for treatment (see Figure 5A,B). Furthermore, the level of *WNT11* mRNA also significantly dropped in pDFs, but exclusively upon exposure to CM-Hyp (see Figure 6B). As reported by Lee et al., the inhibition of the canonical Wnt pathway suppresses epithelial-to-mesenchymal transition (EMT) and inhibits collagen synthesis by irradiated HaCaT cells and human DFs, respectively [50]. These data indicate that silencing of Wnt signaling may abolish dermal fibrosis and reinforce skin homeostasis [50]. Hence, we speculate that the CMs-induced down-regulation of the transcripts of selected Wnt-signaling ligands that has been revealed in our study may offer an alternative approach to chemical inhibitors of the pathway. In addition, this result points out that CMs could be considered for therapeutic purposes. This conclusion, however, is primarily linked with the effect exerted by CM-Hyp on pKERs. It is further supported by the fact that treatment with CM-Hyp increases the mRNA levels of basal-specific *KRT14*, which is a marker of actively proliferating keratinocytes, whereas the transcripts of early differentiation markers (*KRT1*, *KRT10*) were down-regulated.

Regarding the effect of pASCs-primed media on dermal identifiers of wound healing, we found mRNA of *TGFB1*, which is a potent fibrogenic growth factor, was up-regulated in pDFs cultured exclusively in CM-Nor (see Figure 6D). Correspondingly, CM-Nor caused an increase in *WNT10A* and *WNT11* expression levels (see Figure 6A,B). All of the above findings are in agreement with the well-documented existence of signaling crosstalk between the Wnt/β-catenin and Tgfβ1 that was shown to operate in human DFs to cause tissue fibrosis [51,52]. In addition, the mRNA levels of ECM proteins, including *COL1A1* and *FN1*, are elevated in pDFs exposed to CM-Nor. This result endorses our collective observations that CM-Nor, but not CM-Hyp, may favor a pro-fibrotic reaction of skin cells in vitro. On the other hand, the set of ECM proteins, including collagens, are necessary for regular wound healing, whereas their excessive deposition contributes to tissue fibrosis [53]. In our study, we did not examine the balance between ECM proteins’ synthesis and degradation that is essential for establishing skin homeostasis. Moreover, we showed the expression of ECM components exclusively on the mRNA level; therefore, their increase might only suggest a tendency of CM-Nor to induce pDFs toward fibrosis development. Furthermore, coincidentally with the increase in the pro-scarring hallmarks such as *COL1A1* and *TGFB1*, upregulation of anti-fibrotic and pro-regenerative markers (*COL3A1* and *TGFB3*) upon CM-Nor treatment have been observed. Although these data remain ambiguous and mandate further research, they suggest that pDFs possess higher sensitivity to CM-Nor than to CM-Hyp in the context of ECM establishing what might have importance for wound healing resolution.

## 4. Materials and Methods

### 4.1. Cell Isolation and Culture

The pASCs were isolated from the subcutaneous fat of domestic gilts (*Sus scrofa; n* = 10 animals) according to a previously described method [29]. Briefly, the adipose tissue was minced and enzymatically digested with 0.1% collagenase type I (Sigma-Aldrich Co., St. Louis, MO, USA) in phosphate-buffered saline (PBS) with 100 IU/mL penicillin and 100 μg/mL streptomycin (Sigma-Aldrich Co., St. Louis, MO, USA) for 3 h at 37 °C with continuous shaking. Subsequently, the samples were centrifuged at 1200 rpm for 5 min, and recovered cells were cultured in adipose-derived stem cell basal medium (ADSC-BM; Poietics™, Lonza, Walkersville, MD, USA) containing 10% fetal bovine serum (FBS), L-glutamine, and gentamicin/amphotericin B (ADSC-GM SingleQuots^®^; Poietics™ Lonza, Walkersville, MD, USA).

The pKERs and pDFs were isolated from 9-day-old newborn piglets (*n* = 5 animals). Skin samples were sliced using a dermatome (Zimmer^®^ Electric Dermatome; Zimmer Biomet Surgical, Inc., Dover, OH, USA). Skin layers were incubated in 1.6 U/mL dispase (Gibco, Life Technologies Corporation, Grand Island, NY, USA) overnight at 4 °C to separate the epidermis from dermis [47]. The next day, the separated epidermis was digested for 3 min in 0.05% trypsin-EDTA solution (Sigma-Aldrich Co., St. Louis, MO, USA) and filtered through 70-μm strainers (Corning Incorporated, NY, USA) for keratinocyte isolation. The pKERs were collected by a series of three trypsin digestions and filtrations followed by centrifugation at 1200 rpm for 5 min at 37 °C. The cell pellet was suspended and seeded in EpiLife™ medium (Gibco, Life Technologies Corporation, Grand Island, NY, USA) supplemented with EDGS growth supplements (Gibco, Life Technologies Corporation, Grand Island, NY, USA), 10 μg/mL gentamicin, and 0.25 μg/mL amphotericin B (Gibco, Life Technologies Corporation, Grand Island, NY, USA).

For isolation of DFs, dermal slices were minced with scissors and enzymatically digested with 0.2% collagenase type I (Sigma-Aldrich Co., St. Louis, MO, USA) in PBS with 1% penicillin/streptomycin for 90 min at 37 °C. The cell suspension was then filtered through 355-μm mesh and collected by centrifugation (1200 rpm, 5 min). Pelleted cells were suspended and cultured in fibroblast basal medium (FBM^®^; Clonetics^®^, Lonza, Verviers, Belgium) supplemented with 2% FBS, insulin, rhFGF-B, and gentamicin/amphotericin B (FGM™-2 SingleQuots™ Supplement Pack, Clonetics^®^, Lonza, Walkersville, MD, USA). The primary cultured pASCs, pKERs, and pDFs (*p* = 0) were cryopreserved in cryopreservation medium (10% dimethyl sulfoxide (DMSO), 10% DMEM/F12, 80% FBS) and frozen until thawing for individual assays. HUVEC (ScienCell Research Laboratories, Carlsbad, CA, USA) were cultured in EBM™-2 Endothelial Cell Growth Basal Medium (Clonetics^®^, Lonza, Verviers, Belgium) supplemented with EGM™-2 Endothelial SingleQuots™ Kit (Clonetics^®^, Lonza, Walkersville, MD, USA).

Passages 0 or 1 (*p* = 0–1) were used for all experiments involving pASCs, pKERs, pDFs, and HUVEC.

The study protocol was approved by the Local Ethics Committee for Experiments on Animals of the University of Warmia and Mazury (Olsztyn, Poland), no. 67/2018.

### 4.2. Collection of pASC-Conditioned Media Forms (CMs)

The pASCs (*p* = 1; *n* = 7 animals) were plated at a density of 1.5 × 10^5^ cells/cm^2^ in adipose-derived stem cell basal medium (ADSC-BM) containing 10% FBS, L-glutamine, and gentamicin/amphotericin B. After pASCs reached 80–90% confluence, they were washed with PBS and further cultured for 24 h in serum-free ADSC-BM with L-glutamine. For normoxic cultures, pASCs were incubated at 21% O_2_ and 5% CO_2_. For the hypoxia study, pASCs were maintained in a multigas incubator (Panasonic MCO-5M-PE, Panasonic Healthcare Co., Ltd., Japan) supplied with a gas mixture composed of 1% O_2_, 5% CO_2_, and balanced nitrogen. Then, CM-Hyp and CM-Nor were collected and filtered through 0.22-µm filters (TPP Techno Plastic Products AG, Switzerland) to remove cell debris. The CM forms were stored at −80 °C prior to use in assays. To study the effect of CM forms on cell characteristics, 50% concentrations of both CM-Nor and CM-Hyp were prepared by mixing with culture media dedicated to maintaining individual cell types (pKERs, DFs, and HUVEC). The media compositions are presented in Table 1.

### 4.3. Quantification of Specific Protein Levels in CM Forms

The concentrations of VEGF, FGF2, IL-1α, IL-1β, IL-2, IL-4, IL-5, IL-9, IL-10, MCP-1 (monocyte chemoattractant protein -1/CCL2), RANTES (CCL5; C-C motif chemokine ligand 5), and granulocyte macrophage-colony stimulating factor (GM-CSF) in pASCs’ CMs (CM-Nor and CM-Hyp; *n* = 7–9 animals) were determined using human BD™ Cytometric Bead Array (CBA) Flex Sets (BD Biosciences, San Diego, CA, USA) according to the manufacturer’s instructions. Analysis was performed using a BD LSR Fortessa Cell Analyzer flow cytometer (Becton Dickinson and Company, BD Biosciences, San Jose, CA, UAS), BD FACS Diva v6.2 Software (Becton Dickinson, Franklin Lakes, NJ, USA), and FCAP Array v3-Version 3.0.19.2091 (BD Biosciences). The CBA kits’ detection range was 10–2500 pg/mL.

### 4.4. In Vitro Wound Migration Assay

In order to establish the proper protocol of pKERs’ scratch assay, different culture conditions were tested initially. First, the cell density (0.5 × 10^6^ and 2.0 × 10^6^), growth surface (untreated and covered with matrix proteins), and media composition (supplemented with growth factors or with no supplements) were tested (data not shown). Next, we determined the effectiveness of different doses of mitomycin C (100 µg/mL, 10 µg/mL, 1 µg/mL, 100 ng/mL, and 10 ng/mL; Sigma Aldrich Co., St. Louis, MO, USA) and different times of its administration (3 or 1 h) for cell division blockage (Supplementary Figure S1). Accordingly, the most efficient protocol for pKERs’ migration assay was used. The pKERs (*p* = 1; *n* = 5 animals) and pDFs (*p* = 1; *n* = 5 animals) were seeded onto 12-well plates at a density of 2.0 × 10^6^ and 5.0 × 10^5^ cells per well, respectively, and allowed to grow in adequate culture media until they reached confluence. The growth surface was covered with a 1:1 mixture of collagen types I and IV (10 µg/cm^2^; Sigma Aldrich Co., St. Louis, MO, USA) to cultivate pKERs. Monolayers of confluent cultures were pre-treated with mitomycin C (10 μg/mL; 1 h for pKERs and 3 h for pDFs) to prevent cell proliferation. Then, the scratches were made across the center of the well with a 200-μL pipet tip [54]. Cells were washed with PBS and cultured either with CM forms diluted 1:1 in appropriate pKERs’ or pDFs’ culture media alone (see Table 1) or supplemented with human recombinant IL-1β (10 ng/mL; Sigma Aldrich Co., St. Louis, MO, USA) or human recombinant bFGF (10 µg/mL; PeproTech, Inc., Cranbury, NJ, USA). The medium for pDFs was additionally supplemented with 2% FBS. The ADSC-BM mixed with respective pKERs’ or pDFs’ culture media supplemented with 1% penicillin/streptomycin was used as a control. Images were recorded with an Olympus microscope (IX51, Olympus Corporation, Tokyo, Japan) equipped with an Olympus digital camera (XC50, Olympus Corporation, Tokyo, Japan). Wound gaps were measured with ImageJ (SciJava software 1.52a; National Institutes of Health, NIH, Bethesda, MD, USA). The area of scratch closure at 0 h was considered to be 100%. The scratched areas were monitored in three randomly selected fields per sample at 0, 8, 24, 48, and 72 h time points.

### 4.5. Collagen Gel Contraction Assay

Three-dimensional collagen lattices were prepared according to the method previously described [55]. In brief, pDFs’ suspensions (1.0 × 10^5^ cells/400 μL; *n* = 5 animals; *p* = 1) were mixed in 200 μL of a solution of rat tail tendon collagen type I (5 mg/mL, Cultrex, R&D Systems, Minneapolis, MN, USA). Next, 500 μL of the mixture was added to each well of a 24-well plate, neutralized with 5 μL of 1 M NaOH, and allowed to polymerize. Gel matrices were then overlaid with 500 μL of CM forms diluted 1:1 in pDFs’ culture medium (see Table 1). The ADSC-BM medium mixed with pDFs’ media supplemented with 1% penicillin/streptomycin was used as control. The gels were documented with Molecular Imager^®^ Gel Doc™ XR+ Imaging System (Bio-Rad Laboratories, Inc., Hercules, CA, USA), ImageLab 4.1 software 1 (Bio-Rad Laboratories, Inc., Hercules, CA, USA), and the areas were measured with ImageJ (SciJava software 1.52a; National Institutes of Health; NIH, Bethesda, MD, USA). The kinetics of contraction was calculated as the percentage of the initial gel area at time 0, which was considered to be 100%.

### 4.6. RNA Isolation and Real-Time Polymerase Chain Reaction (PCR)

The pKERs (*p* = 0; *n* = 5 animals) and pDFs (*p* = 1; *n* = 4–5 animals) were plated in 6-well plates at a density of 2.0 × 10^6^ or 1.0 × 10^6^ cells per well, respectively, in adequate culture media. Subconfluent (60–70% confluency) cultures were washed with PBS and incubated with CM-Hyp or CM-Nor diluted 1:1 in appropriate culture media (see Table 1) for 24 and 48 h. The ADSC-BM medium mixed with respective KERs’ and DFs’ media supplemented with 1% penicillin/streptomycin was used as a control. Total RNA was extracted using TRIzol^®^ Reagent (Invitrogen by Thermo Fisher Scientific Baltics UAB). Genomic DNA was removed from RNA samples using DNase I Amplification Grade kit (Sigma-Aldrich Co., St. Louis, MO, USA). Synthesis of complementary DNA with 1000 ng of RNA was performed using High-Capacity cDNA Reverse Transcription Kits with RNase Inhibitor (Applied Biosystems by Thermo Fisher Scientific Baltics UAB) according to the manufacturer’s specifications. Expression of specific mRNA was quantified with TaqMan Gene Expression Assays (Applied Biosystems by Thermo Fisher Scientific Baltics UAB) on an ABI ViiA™ 7-sequence detection system (Applied Biosystems by Life Technologies, Singapore). Gene names and primer-probe set information are presented in Supplementary Table S1. All results were normalized to *HPRT1* expression as endogenous control using the PCR Miner algorithm [56]. 

### 4.7. Protein Isolation and Western Blot

The pDFs (*p* = 1; *n* = 4–5 animals) were plated in 6-well plates at a density of 2.0 × 10^6^ in adequate culture media. Subconfluent (60–70% confluency) cultures were washed with PBS and incubated with CMs diluted 1:1 in pDFs’ culture medium (see Table 1) for 48 h. The ADSC-BM medium mixed with DFs’ medium supplemented with 1% penicillin/streptomycin was used as control. Next, the cells were homogenized in a RIPA buffer (Thermo Scientific, Rockford, IL, USA) containing a protease inhibitor cocktail (Sigma-Aldrich Co., St. Louis, MO, USA). Twenty-five micrograms of proteins per sample were separated on 12% SDS-PAGE gels and blotted onto polyvinyl difluoride (PVDF) membranes. Membranes were incubated separately with anti-αSMA (1:2000; ab15734, Abcam, Cambridge, MA, USA) and anti-β-actin (1:1000, ab8226, Abcam, Cambridge, MA, USA) primary antibodies. After incubation with fluorescent anti-mouse (Cy5.5, 1:, Rockland Immunochemicals, Inc., Limerick, PA, USA) and anti-rabbit (IRDye 800, 1:5000, Rockland Immunochemicals, Inc., Limerick, PA, USA) secondary antibodies, bands were visualized using the ChemiDoc™ Imaging System (Bio-Rad Laboratories, Inc., Hercules, CA, USA).

### 4.8. Angiogenesis Assay

HUVEC (*p* = 1; *n* = 4 replicates) were seeded at 2.5 × 10^4^ viable cells/well in a µ-Plate Angiogenesis 96-well plate (Ibidi GmbH, Germany) coated with 10 µL Geltrex™ Reduced Growth Factor Basement Membrane (Gibco, Life Technologies Corporation, Grand Island, NY, USA) per well. Cells were incubated with CM forms diluted 1:1 in Endothelial Cell Growth Basal Medium-2 (EBM™-2; see Table 1). The ADSC-BM medium diluted 1:1 in EBM™-2 and supplemented with 1% penicillin/streptomycin was used as a control. Images depicting tube formation were obtained using a Zeiss Axio Observer.Z1 (Carl Zeiss Microscopy GmbH, Germany) microscope equipped with ZEN2.6 (Blue edition) software (Carl Zeiss Microscopy GmbH, Germany). Formation of capillary-like structures was monitored at 0, 7, 10, and 16 h time points. Images were analyzed with CellSens Dimension software (Olympus Corporation, Tokyo, Japan) with a TruAI neural network module (Olympus Corporation, Tokyo, Japan). First, the neural network was adapted to correctly detect angiogenic loops. Next, the network was executed to assess the number of loops and their area (µm^2^) per image. Based on their size, loops were divided into three groups, namely, small (2000–10,000 µm^2^), medium (10,000–50,000 µm^2^), and large (>50,000 µm^2^).

### 4.9. Statistical Analysis

Statistical analyses of CMs secretome composition and its effect on mRNA expression and HUVEC functionality were performed using GraphPad PRISM, version 8.4.3 software (GraphPad Software Inc., San Diego, CA, USA). A paired *t*-test was performed to examine the levels of VEGF, bFGF, and MCP-1 secreted into CMs. Two-way analysis of variance (ANOVA) followed by Tukeys’s or Bonferroni’s post hoc tests were used to analyze (1) specific mRNA expression in pKERs and pDFs exposed to CMs and (2) HUVEC networking formation. Data are expressed as mean ± standard deviation (SD). A value of *p* < 0.05 was considered statistically significant.

Migratory abilities of pKERs and pDFs cultured in CMs and pDFs contractility were analyzed using linear mixed-effects models in which individuals were included as random intercept and different factors and their interactions were included as fixed effects. In cases of insignificant random effects, linear models were used instead. Least-square means (lsmeans) were calculated and compared based on models that were valid (the Kenward–Roger method for degrees of freedom was used for mixed-effect models; a Tukey *p*-value adjustment was used where appropriate). The level of significance was assumed to be *p* = 0.05, with statistically significant results for the levels *p* = 0.01 and *p* = 0.001 mentioned where appropriate. All calculations referring to the cell migration and contractility were performed in R (ver. 4.0.2) using packages lme4 (ver. 1.1) and lsmeans (ver. 2.25). Statistical analysis of cell migration and contraction was performed by Biostat, Poland (https://www.biostat.com.pl/index_en.php) (access date 21 May 2021).

## 5. Conclusions

The results obtained in the present study indicate that hypoxia (1% O_2_) exerts a moderate effect on pASCs’ secretome. The impact of both CM-Nor and CM-Hyp on the functional characteristics and the molecular signature of skin cells in vitro clearly demonstrates that the bioactivity of both CMs is cell-specific and contrasting in terms of their effect on markers attributed to wound healing expressed by pDFs. To the best of our knowledge, this is the first report demonstrating the validation of a secretome of pig ASCs in the context of its future use for therapeutic purposes. Such ASC-related therapies suggest the promise of a cutaneous wound healing process, but in vitro testing is needed to ensure their safety and reliability before in vivo preclinical translation.

## Figures and Tables

**Table 1:** Media composition.

Cell Type	Media Dilution 1:1	
	Cell-Specific Culture Media Used for CMs Dilution	pASCs Culture Medium Used for CM Production
pKERs	EpiLife™ (Gibco, Life Technologies Corporation, Grand Island, NY, USA)	ADSC-BM Adipose-Derived Stem Cell Basal Medium (Poietics™, Lonza, Walkersville, MD, USA)
pDFs	FBM^®^ Fibroblast Basal Medium (Clonetics^®^, Lonza, Verviers, Belgium)	
HUVEC	EBM™-2 Endothelial Cell Growth Basal Medium (Clonetics^®^, Lonza, Verviers, Belgium)	

## Data Availability

The data presented in this study are available in the article and in supplementary material here.

## Supplementary Materials

The following are available online at https://www.mdpi.com/article/10.3390/ijms22115469/s1.

## Abbreviations

ASCs	adipose-derived stem cells
bFGF	basic fibroblasts growth factor
BM-MSCs	bone marrow mesenchymal stem cells
CM	conditioned medium
CM-Hyp	CM collected from pASCs cultured in hypoxia
CM-Nor	CM collected from pASCs cultured in normoxia
Col1α1	collagen type I alpha 1 chain
Col3α1	collagen type III alpha 1 chain
CTGF (CCN2)	connective tissue growth factors (cellular communication network factor 2)
DMEM/F12	Dulbecco’s Modified Eagle Medium/Nutrient Mixture F-12
DMSO	dimethyl sulfoxide
ECM	extracellular matrix
FBS	fetal bovine serum
FN1	fibronectin 1
Foxn1	forkhead box N1 transcription factor
GM-CSF	granulocyte macrophage-colony stimulating factor
HaCaT	human keratinocyte line
HBO	hyperbaric oxygen therapy
HDF	human dermal fibroblasts
HDLECs	dermal lymphatic endothelial cells
HGF	hepatocyte growth factor
HUVEC	human umbilical vein endothelial cells
IGF-1	insulin-like growth factor-1
IL-1β	interleukin 1 beta
IVL	involucrin
KRT 1, 6a, 10, 14, 17	keratins 1, 6a, 10, 14, 17
MCP-1 (CCL2)	monocyte chemoattractant protein -1 (C-C motif chemokine ligand 2)
MMP-1	matrix metalloproteinase 1
NPWT	negative pressure wound therapy
pASCs	pig adipose-derived stem cells
PCR	polymerase chain reaction
pDFs	pig dermal fibroblasts
pKERs	pig keratinocytes
RANTES (CCL5)	C-C motif chemokine ligand 5
Tgfβ1	transforming growth factor beta 1
Tgfβ3	transforming growth factor beta 3
TNC	tenascin C
TNF-α	tumor necrosis growth factor alpha
VEGF	vascular endothelial growth factor
WNT10A	Wingless family member 10a
WNT11	Wingless family member 11
αSMA	smooth muscle actin alpha

## References

[1] Lorenz H.P., Adzick N.S. (1993). Scarless Skin Wound Repair in the Fetus. West. J. Med..

[2] Gawronska-Kozak B. (2004). Regeneration in the ears of immunodeficient mice: Identification and lineage analysis of mesenchymal stem cells. Tissue Eng..

[3] Gawronska-Kozak B. (2011). Scarless skin wound healing in FOXN1 deficient (nude) mice is associated with distinctive matrix metalloproteinase expression. Matrix Biol..

[4] Gawronska-Kozak B., Bogacki M., Rim J.S., Monroe W.T., Manuel J.A. (2006). Scarless skin repair in immunodeficient mice. Wound Repair Regen..

[5] Seifert A.W., Kiama S.G., Seifert M.G., Goheen J.R., Palmer T.M., Maden M. (2012). Skin shedding and tissue regeneration in African spiny mice (Acomys). Nature.

[6] Gawronska-Kozak B., Grabowska A., Kur-Piotrowska A., Kopcewicz M. (2016). Foxn1 Transcription Factor Regulates Wound Healing of Skin through Promoting Epithelial-Mesenchymal Transition. PLoS ONE.

[7] Lo D.D., Zimmermann A.S., Nauta A., Longaker M.T., Lorenz H.P. (2012). Scarless fetal skin wound healing update. Birth Defects Res. Part C Embryo Today.

[8] Chen W., Fu X., Ge S., Sun T., Zhou G., Jiang D., Sheng Z. (2005). Ontogeny of expression of transforming growth factor-beta and its receptors and their possible relationship with scarless healing in human fetal skin. Wound Repair Regen..

[9] Lovvorn H.N., Cheung D.T., Nimni M.E., Perelman N., Estes J.M., Adzick N.S. (1999). Relative distribution and crosslinking of collagen distinguish fetal from adult sheep wound repair. J. Pediatr. Surg..

[10] Merkel J.R., DiPaolo B.R., Hallock G.G., Rice D.C. (1988). Type I and type III collagen content of healing wounds in fetal and adult rats. Proc. Soc. Exp. Biol. Med..

[11] Hinz B. (2007). Formation and function of the myofibroblast during tissue repair. J. Investig. Dermatol..

[12] Bastakoty D., Saraswati S., Cates J., Lee E., Nanney L.B., Young P.P. (2015). Inhibition of Wnt/beta-catenin pathway promotes regenerative repair of cutaneous and cartilage injury. FASEB J..

[13] Bukowska J., Walendzik K., Kopcewicz M., Cierniak P., Gawronska-Kozak B. (2021). Wnt signaling and the transcription factor Foxn1 contribute to cutaneous wound repair in mice. Connect. Tissue Res..

[14] Strong A.L., Bowles A.C., MacCrimmon C.P., Frazier T.P., Lee S.J., Wu X., Katz A.J., Gawronska-Kozak B., Bunnell B.A., Gimble J.M. (2015). Adipose stromal cells repair pressure ulcers in both young and elderly mice: Potential role of adipogenesis in skin repair. Stem Cells Transl. Med..

[15] Jiang X., Zhang H., Teng M. (2016). Effectiveness of Autologous Stem Cell Therapy for the Treatment of Lower Extremity Ulcers: A Systematic Review and Meta-Analysis. Medicine.

[16] Marino G., Moraci M., Armenia E., Orabona C., Sergio R., de Sena G., Capuozzo V., Barbarisi M., Rosso F., Giordano G. (2013). Therapy with autologous adipose-derived regenerative cells for the care of chronic ulcer of lower limbs in patients with peripheral arterial disease. J. Surg. Res..

[17] Nie C., Yang D., Xu J., Si Z., Jin X., Zhang J. (2011). Locally administered adipose-derived stem cells accelerate wound healing through differentiation and vasculogenesis. Cell Transplant..

[18] Feng J., Mineda K., Wu S.H., Mashiko T., Doi K., Kuno S., Kinoshita K., Kanayama K., Asahi R., Sunaga A. (2017). An injectable non-cross-linked hyaluronic-acid gel containing therapeutic spheroids of human adipose-derived stem cells. Sci. Rep..

[19] Wang L., Hu L., Zhou X., Xiong Z.H., Zhang C.G., Shehada H.M.A., Hu B., Song J.L., Chen L.L. (2017). Exosomes secreted by human adipose mesenchymal stem cells promote scarless cutaneous repair by regulating extracellular matrix remodelling. Sci. Rep..

[20] Ahmadzadeh N., Robering J.W., Kengelbach-Weigand A., Al-Abboodi M., Beier J.P., Horch R.E., Boos A.M. (2020). Human adipose-derived stem cells support lymphangiogenesis in vitro by secretion of lymphangiogenic factors. Exp. Cell Res..

[21] Kilroy G.E., Foster S.J., Wu X., Ruiz J., Sherwood S., Heifetz A., Ludlow J.W., Stricker D.M., Potiny S., Green P. (2007). Cytokine profile of human adipose-derived stem cells: Expression of angiogenic, hematopoietic, and pro-inflammatory factors. J. Cell. Physiol..

[22] Nakagami H., Maeda K., Morishita R., Iguchi S., Nishikawa T., Takami Y., Kikuchi Y., Saito Y., Tamai K., Ogihara T. (2005). Novel autologous cell therapy in ischemic limb disease through growth factor secretion by cultured adipose tissue-derived stromal cells. Arterioscler. Thromb. Vasc. Biol..

[23] Sadat S., Gehmert S., Song Y.H., Yen Y., Bai X., Gaiser S., Klein H., Alt E. (2007). The cardioprotective effect of mesenchymal stem cells is mediated by IGF-I and VEGF. Biochem. Biophys. Res. Commun..

[24] Park S.R., Kim J.W., Jun H.S., Roh J.Y., Lee H.Y., Hong I.S. (2018). Stem Cell Secretome and Its Effect on Cellular Mechanisms Relevant to Wound Healing. Mol. Ther..

[25] Cooper D.R., Wang C., Patel R., Trujillo A., Patel N.A., Prather J., Gould L.J., Wu M.H. (2018). Human Adipose-Derived Stem Cell Conditioned Media and Exosomes Containing MALAT1 Promote Human Dermal Fibroblast Migration and Ischemic Wound Healing. Adv. Wound Care.

[26] Kim M.H., Wu W.H., Choi J.H., Kim J., Jun J.H., Ko Y., Lee J.H. (2018). Galectin-1 from conditioned medium of three-dimensional culture of adipose-derived stem cells accelerates migration and proliferation of human keratinocytes and fibroblasts. Wound Repair Regen..

[27] Hsiao S.T., Lokmic Z., Peshavariya H., Abberton K.M., Dusting G.J., Lim S.Y., Dilley R.J. (2013). Hypoxic conditioning enhances the angiogenic paracrine activity of human adipose-derived stem cells. Stem Cells Dev..

[28] Lee E.Y., Xia Y., Kim W.S., Kim M.H., Kim T.H., Kim K.J., Park B.S., Sung J.H. (2009). Hypoxia-enhanced wound-healing function of adipose-derived stem cells: Increase in stem cell proliferation and up-regulation of VEGF and bFGF. Wound Repair Regen..

[29] Bukowska J., Slowinska M., Cierniak P., Kopcewicz M., Walendzik K., Frazier T., Gawronska-Kozak B. (2020). The effect of hypoxia on the proteomic signature of pig adipose-derived stromal/stem cells (pASCs). Sci. Rep..

[30] Jokela T., Oikari S., Takabe P., Rilla K., Karna R., Tammi M., Tammi R. (2015). Interleukin-1beta-induced Reduction of CD44 Ser-325 Phosphorylation in Human Epidermal Keratinocytes Promotes CD44 Homomeric Complexes, Binding to Ezrin, and Extended, Monocyte-adhesive Hyaluronan Coats. J. Biol. Chem..

[31] Sanmiguel J.C., Olaru F., Li J., Mohr E., Jensen L.E. (2009). Interleukin-1 regulates keratinocyte expression of T cell targeting chemokines through interleukin-1 receptor associated kinase-1 (IRAK1) dependent and independent pathways. Cell. Signal..

[32] Kanazawa S., Fujiwara T., Matsuzaki S., Shingaki K., Taniguchi M., Miyata S., Tohyama M., Sakai Y., Yano K., Hosokawa K. (2010). bFGF regulates PI3-kinase-Rac1-JNK pathway and promotes fibroblast migration in wound healing. PLoS ONE.

[33] Schreier T., Degen E., Baschong W. (1993). Fibroblast migration and proliferation during in vitro wound healing. A quantitative comparison between various growth factors and a low molecular weight blood dialysate used in the clinic to normalize impaired wound healing. Res. Exp. Med..

[34] Chujo S., Shirasaki F., Kondo-Miyazaki M., Ikawa Y., Takehara K. (2009). Role of connective tissue growth factor and its interaction with basic fibroblast growth factor and macrophage chemoattractant protein-1 in skin fibrosis. J. Cell. Physiol..

[35] Stubbs S.L., Hsiao S.T., Peshavariya H.M., Lim S.Y., Dusting G.J., Dilley R.J. (2012). Hypoxic preconditioning enhances survival of human adipose-derived stem cells and conditions endothelial cells in vitro. Stem Cells Dev..

[36] Hu X.Y., Yu S.P., Fraser J.L., Lu Z.Y., Ogle M.E., Wang J.A., Wei L. (2008). Transplantation of hypoxia-preconditioned mesenchymal stem cells improves infarcted heart function via enhanced survival of implanted cells and angiogenesis. J. Thorac. Cardiovasc. Surg..

[37] Tajiri N., Acosta S.A., Shahaduzzaman M., Ishikawa H., Shinozuka K., Pabon M., Hernandez-Ontiveros D., Kim D.W., Metcalf C., Staples M. (2014). Intravenous transplants of human adipose-derived stem cell protect the brain from traumatic brain injury-induced neurodegeneration and motor and cognitive impairments: Cell graft biodistribution and soluble factors in young and aged rats. J. Neurosci..

[38] Chen L., Xu Y., Zhao J., Zhang Z., Yang R., Xie J., Liu X., Qi S. (2014). Conditioned medium from hypoxic bone marrow-derived mesenchymal stem cells enhances wound healing in mice. PLoS ONE.

[39] Shi H.X., Lin C., Lin B.B., Wang Z.G., Zhang H.Y., Wu F.Z., Cheng Y., Xiang L.J., Guo D.J., Luo X. (2013). The anti-scar effects of basic fibroblast growth factor on the wound repair in vitro and in vivo. PLoS ONE.

[40] Xie J.L., Bian H.N., Qi S.H., Chen H.D., Li H.D., Xu Y.B., Li T.Z., Liu X.S., Liang H.Z., Xin B.R. (2008). Basic fibroblast growth factor (bFGF) alleviates the scar of the rabbit ear model in wound healing. Wound Repair Regen..

[41] DiPietro L.A., Polverini P.J., Rahbe S.M., Kovacs E.J. (1995). Modulation of JE/MCP-1 expression in dermal wound repair. Am. J. Pathol..

[42] Gibran N.S., Ferguson M., Heimbach D.M., Isik F.F. (1997). Monocyte chemoattractant protein-1 mRNA expression in the human burn wound. J. Surg. Res..

[43] Krzyszczyk P., Schloss R., Palmer A., Berthiaume F. (2018). The Role of Macrophages in Acute and Chronic Wound Healing and Interventions to Promote Pro-wound Healing Phenotypes. Front. Physiol..

[44] Wood S., Jayaraman V., Huelsmann E.J., Bonish B., Burgad D., Sivaramakrishnan G., Qin S., DiPietro L.A., Zloza A., Zhang C. (2014). Pro-inflammatory chemokine CCL2 (MCP-1) promotes healing in diabetic wounds by restoring the macrophage response. PLoS ONE.

[45] Riis S., Newman R., Ipek H., Andersen J.I., Kuninger D., Boucher S., Vemuri M.C., Pennisi C.P., Zachar V., Fink T. (2017). Hypoxia enhances the wound-healing potential of adipose-derived stem cells in a novel human primary keratinocyte-based scratch assay. Int. J. Mol. Med..

[46] Grellner W., Georg T., Wilske J. (2000). Quantitative analysis of proinflammatory cytokines (IL-1beta, IL-6, TNF-alpha) in human skin wounds. Forensic Sci. Int..

[47] Ponce L., Heintz F., Schäfer I., Klusch A., Holloschi A., Schmelz M., Petersen M., Hafner M. (2017). Isolation and Cultivation of Primary Keratinocytes from Piglet Skin for Compartmentalized Co-culture with Dorsal Root Ganglion Neurons. J. Cell. Biotechnol..

[48] Xuan Y.H., Huang B.B., Tian H.S., Chi L.S., Duan Y.M., Wang X., Zhu Z.X., Cai W.H., Zhu Y.T., Wei T.M. (2014). High-glucose inhibits human fibroblast cell migration in wound healing via repression of bFGF-regulating JNK phosphorylation. PLoS ONE.

[49] Beyer C., Schramm A., Akhmetshina A., Dees C., Kireva T., Gelse K., Sonnylal S., de Crombrugghe B., Taketo M.M., Distler O. (2012). Beta-catenin is a central mediator of pro-fibrotic Wnt signaling in systemic sclerosis. Ann. Rheum. Dis..

[50] Lee D.W., Lee W.J., Cho J., Yun C.O., Roh H., Chang H.P., Roh T.S., Lee J.H., Lew D.H. (2020). Inhibition of Wnt signaling pathway suppresses radiation-induced dermal fibrosis. Sci. Rep..

[51] Akhmetshina A., Palumbo K., Dees C., Bergmann C., Venalis P., Zerr P., Horn A., Kireva T., Beyer C., Zwerina J. (2012). Activation of canonical Wnt signalling is required for TGF-beta-mediated fibrosis. Nat. Commun..

[52] Sato M. (2006). Upregulation of the Wnt/beta-catenin pathway induced by transforming growth factor-beta in hypertrophic scars and keloids. Acta Derm. Venereol..

[53] Ehrlich H.P., Krummel T.M. (1996). Regulation of wound healing from a connective tissue perspective. Wound Repair Regen..

[54] Gawronska-Kozak B., Kirk-Ballard H. (2013). Cyclosporin A reduces matrix metalloproteinases and collagen expression in dermal fibroblasts from regenerative FOXN1 deficient (nude) mice. Fibrogenes. Tissue Repair.

[55] Bukowska J., Kopcewicz M., Kur-Piotrowska A., Szostek-Mioduchowska A.Z., Walendzik K., Gawronska-Kozak B. (2018). Effect of TGFbeta1, TGFbeta3 and keratinocyte conditioned media on functional characteristics of dermal fibroblasts derived from reparative (Balb/c) and regenerative (Foxn1 deficient; nude) mouse models. Cell Tissue Res..

[56] Zhao S., Fernald R.D. (2005). Comprehensive algorithm for quantitative real-time polymerase chain reaction. J. Comput. Biol..

