# Supplementary material for: Effect of Pig-Adipose-Derived Stem Cells’ Conditioned Media on Skin Wound-Healing Characteristics In Vitro
Source: Int J Mol Sci. 2021 May 22;22(11):5469. doi: 10.3390/ijms22115469 (PMC8196863; doi:10.3390/ijms22115469)
Supplement: Supplementary file 1 [file ijms-22-05469-s001.zip › ijms-1202247-supplementary.pdf]

Effect of Pig-Adipose-Derived Stem Cells' Conditioned Media on Skin Wound-Healing Characteristics In Vitro

Joanna Wiśniewska, Magda Słyszewska, Karolina Stałanowska, Katarzyna Walendzik,

Marta Kopcewicz, Sylwia Machcińska, Barbara Gawrońska-Kozak

Supplementary Table S1: Gene names and primer-probe sets used in real-time PCR.

Inventoried TaqMan® Gene Expression Assays		
**Gene symbol**	**Gene name**	**Primer and probe sets ID**
CCN2 (CTGF)	Cellular communication network factor 2 (Connective tissue growth factor)	Ss03392397_m1
COL1A1	Collagen type I alpha 1 chain	Ss03373340_m1
COL3A1	Collagen type III alpha 1 chain	Ss04323772_g1
FN1	Fibronectin 1	Ss03373673_m1
IVL	Involucrin	Ss03822369_s1
KRT1	Keratin 1	Ss03372954_m1
KRT6A	Keratin 6a	Ss06938590_s1
TGFB1	Transforming growth factor beta 1	Ss04955543_m1
TGFB3	Transforming growth factor beta 1	Ss03394351_m1
TNC	Tenascin C	Ss03394416_m1
HPRT1	Hypoxanthine phosphoribosyltransferase 1	Ss03388274

Made to order TaqMan® Gene Expression Assays			
**Gene symbol**	**Gene name**	**Primer/probe**	**Sequence 5′→3′**
AXIN2	Axin2	Forward primer<br>Reverse primer<br>Probe	GAGGGAGAAATGCGTGGATA<br>GGTTTCAGCTGCTTGGAGAC<br>ACTTCTGGTTTGCCTGCAAT
KRT10	Keratin 10	Forward primer	CCCAACTAGCCCTGAAACAA<br>GTATTCGGCATTCTGGCACT

		Reverse primer<br>Probe	CTACTGTGTGCAGCTCGCTC
KRT17	Keratin 17	Forward primer<br>Reverse primer<br>Probe	GAACCACGAGGAGGAGATGA<br>CTCCGTCTTGCTGAAGAACC<br>GTACGAGAAGATGGCCGAGA
WNT10A	Wingless family member 10A	Forward primer<br>Reverse primer<br>Probe	ACGAGTGCCAACACCAGTTC<br>AGGCACTCTCTCGGAAACCT<br>GCGCTGGAACTGCTCTAGTC
WNT11	Wingless family member 11	Forward primer<br>Reverse primer<br>Probe	GAGCTCATGCACACTGTCGT<br>TCTCTCCAGGTCAAGCAGGT<br>ACTGCTCCTCCATCGAGCT

Supplementary Table S2: Presentation of statistical analysis depicting the effect of different media (Ctrl medium, CM-Nor, or CM-Hyp) on the migration of pKERs over the time course (statistics refer to Figure 2A in the main body of manuscript).

Hours [h]	Group	lsmean	lower.CL	upper.CL	SE	df	8	24	48
**0**	**Ctrl medium**	100,0	83,1	116,9	7,96	15,4	0,94	0,935	**0,021**
**8**	**Ctrl medium**	95,2	78,3	112,1	7,96	15,4		0,656	**0,004**
**24**	**Ctrl medium**	105,0	88,0	121,9	7,96	15,4			0,084
**48**	**Ctrl medium**	125,5	108,6	142,5	7,96	15,4			
**0**	**CM-Nor**	100,0	83,1	116,9	7,96	15,4	0,954	0,927	0,991
**8**	**CM-Nor**	95,6	78,7	112,6	7,96	15,4		1,000	0,996
**24**	**CM-Nor**	94,8	77,9	111,7	7,96	15,4			0,988
**48**	**CM-Nor**	97,5	80,6	114,5	7,96	15,4			
**0**	**CM-Hyp**	100,0	83,1	116,9	7,96	15,4	0,228	**0,043**	0,133
**8**	**CM-Hyp**	83,7	66,7	100,6	7,96	15,4		0,856	0,992
**24**	**CM-Hyp**	77,0	60,0	93,9	7,96	15,4			0,956
**48**	**CM-Hyp**	81,3	64,3	98,2	7,96	15,4			

Supplementary Table S3: Presentation of statistical analysis depicting the effect of CM-Nor and CM-Nor +IL-1β on pKERs migration over the time course (statistics refer to Figure 2B in the main body of manuscript).

	**Estimate**	**Std. Error**	**t value**	**Pr(>ǀtǀ)**
**(Intercept)**	100,000	5,47	18,279	**<0,001**
**Group CM-Nor+IL-1β**	0,000	7,74	0,000	1,000
**Hours 8**	-4,374	7,74	-0,565	0,576
**Hours 24**	-5,185	7,74	-0,670	0,508
**Hours 8**	-2,466	7,74	-0,319	0,752
**Group CM-Nor+IL-1β: Hours 8**	0,032	10,94	0,003	0,998
**Group CM-Nor+IL-1β: Hours 24**	-0,549	10,94	-0,050	0,960
**Group CM-Nor+IL-1β: Hours 48**	17,557	10,94	1,605	0,118
**Observations**	**Residual Std. Error**	R^2	**Adjusted** R^2	
40	12,2	0,254	0,09	

Supplementary Table S4: Presentation of statistical analysis depicting the effect of CM-Hyp and CM-Hyp +IL-1β on pKERs migration over the time course (statistics refer to Figure 2C in the main body of manuscript).

Hours [h]	Group	lsmean	lower.CL	upper.CL	SE	df	8	24	48
**0**	**CM-Hyp**	100,0	82,1	117,9	8,54	19,4	0,384	0,126	0,268
**8**	**CM-Hyp**	83,7	65,8	101,5	8,54	19,4		0,909	0,995
**24**	**CM-Hyp**	77,0	59,1	94,8	8,54	19,4			0,973
**48**	**CM-Hyp**	81,3	63,4	99,1	8,54	19,4			
**0**	**CM-Hyp +IL-1β**	100,0	82,1	117,9	8,54	19,4	0,211	0,377	0,998
**8**	**CM-Hyp + IL-1β**	79,8	62,0	97,7	8,54	19,4		0,982	0,151
**24**	**CM-Hyp + IL-1β**	83,5	65,7	101,4	8,54	19,4			0,286
**48**	**CM-Hyp + IL-1β**	101,9	84,0	119,7	8,54	19,4			

Supplementary Table S5: Presentation of statistical analysis depicting the effect of different media (Ctrl medium, CM-Nor, or CM-Hyp) on pDFs migration over the time course (statistics refer to Figure 3A in the main body of manuscript).

Hours [h]	Group	lsmean	lower.CL	upper.CL	SE	df	24	48	72
**0**	**Ctrl medium**	100,00	93,08	106,92	3,35	23,9	**<0,001**	**<0,001**	**<0,001**
**24**	**Ctrl medium**	9,47	2,55	16,39	3,35	23,9		0,271	0,093
**48**	**Ctrl medium**	2,20	-4,72	9,12	3,35	23,9			0,944
**72**	**Ctrl medium**	0,00	-6,92	6,92	3,35	23,9			
**0**	**CM-Nor**	100,00	93,08	106,92	3,35	23,9	**<0,001**	**<0,001**	**<0,001**
**24**	**CM-Nor**	25,31	18,39	32,23	3,35	23,9		**<0,001**	**<0,001**
**48**	**CM-Nor**	4,58	-2,34	11,50	3,35	23,9			0,656
**72**	**CM-Nor**	0,00	-6,92	6,92	3,35	23,9			
**0**	**CM-Hyp**	100,00	93,08	106,92	3,35	23,9	**<0,001**	**<0,001**	**<0,001**
**24**	**CM-Hyp**	34,23	27,31	41,15	3,35	23,9		**<0,001**	**<0,001**
**48**	**CM-Hyp**	15,41	8,49	22,33	3,35	23,9			**0,002**
**72**	**CM-Hyp**	0,00	-6,92	6,92	3,35	23,9			

Supplementary Table S6: Presentation of statistical analysis depicting the effect of CM-Nor and CM-Nor +bFGF on pDFs migration over the time course (statistics refer to Figure 3B in the main body of manuscript).

Hours [h]	Group	lsmean	lower.CL	upper.CL	SE	df	24	48	72
**0**	**CM-Nor**	100,00	92,96	107,04	3,46	32	**<0,001**	**<0,001**	**<0,001**
**24**	**CM-Nor**	25,31	18,27	32,35	3,46	32		**<0,001**	**<0,001**
**48**	**CM-Nor**	4,58	-2,46	11,62	3,46	32			0,785
**72**	**CM-Nor**	0,00	-7,04	7,04	3,46	32			
**0**	**CM-Nor+bFGF**	100,00	92,96	107,04	3,46	32	**<0,001**	**<0,001**	**<0,001**
**24**	**CM-Nor+bFGF**	18,89	11,85	25,94	3,46	32		**0,003**	**0,003**
**48**	**CM-Nor+bFGF**	0,00	-7,04	7,04	3,46	32			1,000
**72**	**CM-Nor+bFGF**	0,00	-7,04	7,04	3,46	32			

Supplementary Table S7: Presentation of statistical analysis depicting the effect of CM-Hyp and CM-Hyp +bFGF on pDFs migration over the time course (statistics refer to Figure 3C in the main body of manuscript).

Hours [h]	Group	lsmean	lower.CL	upper.CL	SE	df	24	48	72
**0**	**CM-Hyp**	100,0	93,70	106,30	3,09	32	**<0,001**	**<0,001**	**<0,001**
**24**	**CM-Hyp**	34,2	27,93	40,52	3,09	32		**0,001**	**<0,001**
**48**	**CM-Hyp**	15,4	9,11	21,70	3,09	32			**0,008**
**72**	**CM-Hyp**	0,0	-6,29	6,29	3,09	32			
**0**	**CM-Hyp+bFGF**	100,0	93,70	106,30	3,09	32	**<0,001**	**<0,001**	**<0,001**
**24**	**CM-Hyp+bFGF**	17,7	11,43	24,02	3,09	32		**0,002**	**0,002**
**48**	**CM-Hyp+bFGF**	0,0	-6,29	6,29	3,09	32			1,000
**72**	**CM-Hyp+bFGF**	0,0	-6,29	6,29	3,09	32			

Supplementary Table S8: Presentation of statistical analysis depicting the effect of different media (Ctrl medium, CM-Nor, or CM-Hyp) on pDFs contractility over the time course (statistics refer to Figure 4A in the main body of manuscript).

Day	Group	lsmean	lower.CL	upper.CL	SE	df	1	2	3	4	5	6	7	8	9
**0**	**Ctrl medium**	100,0	93,3	106,7	3,23	21,2	**<0,001**	**<0,001**	**<0,001**	**<0,001**	**<0,001**	**<0,001**	**<0,001**	**<0,001**	**<0,001**
**1**	**Ctrl medium**	84,1	77,4	90,8	3,23	21,2		0,289	0,106	0,2	**0,036**	**<0,001**	**<0,001**	**<0,001**	**<0,001**
**2**	**Ctrl medium**	75,3	68,5	82,0	3,23	21,2			1,000	1,000	0,997	0,453	0,252	0,325	0,205
**3**	**Ctrl medium**	73,6	66,9	80,4	3,23	21,2				1,000	1,000	0,746	0,517	0,612	0,448
**4**	**Ctrl medium**	74,6	67,9	81,3	3,23	21,2					1,000	0,573	0,347	0,433	0,289
**5**	**Ctrl medium**	72,2	65,5	78,9	3,23	21,2						0,924	0,768	0,842	0,705
**6**	**Ctrl medium**	66,9	59,8	74,0	3,48	27,2							1,000	1,000	1,000

**7**	**Ctrl medium**	65,6	58,5	72,7	3,48	27,2								1,000	1,000
**8**	**Ctrl medium**	66,1	59,0	73,3	3,48	27,2									1,000
**9**	**Ctrl medium**	65,2	58,1	72,4	3,48	27,2									
**0**	**CM-Nor**	100,0	93,3	106,7	3,23	21,2	**<0,001**	**<0,001**	**<0,001**	**<0,001**	**<0,001**	**<0,001**	**<0,001**	**<0,001**	**<0,001**
**1**	**CM-Nor**	75,8	69,1	82,5	3,23	21,2		0,625	0,211	0,104	**0,004**	**0,001**	**<0,001**	**<0,001**	**<0,001**
**2**	**CM-Nor**	68,8	62,1	75,5	3,23	21,2			1,000	0,992	0,531	0,254	0,142	**0,002**	**<0,001**
**3**	**CM-Nor**	66,4	59,6	73,1	3,23	21,2				1,000	0,918	0,66	0,473	**0,02**	**0,003**
**4**	**CM-Nor**	65,3	58,5	72,0	3,23	21,2					0,982	0,834	0,672	**0,046**	**0,009**
**5**	**CM-Nor**	61,3	54,6	68,0	3,23	21,2						1,000	0,998	0,452	0,163
**6**	**CM-Nor**	59,1	52,0	66,3	3,48	27,2							1,000	0,863	0,544
**7**	**CM-Nor**	58,1	51,0	65,3	3,48	27,2								0,951	0,72
**8**	**CM-Nor**	52,9	45,8	60,1	3,48	27,2									1,000
**9**	**CM-Nor**	50,9	43,7	58,0	3,48	27,2									
**0**	**CM-Hyp**	100,0	93,3	106,7	3,23	21,2	**<0,001**	**<0,001**	**<0,001**	**<0,001**	**<0,001**	**<0,001**	**<0,001**	**<0,001**	**<0,001**
**1**	**CM-Hyp**	72,7	65,9	79,4	3,23	21,2		0,504	0,078	**0,028**	**0,04**	**0,012**	**0,007**	**<0,001**	**<0,001**
**2**	**CM-Hyp**	65,1	58,3	71,8	3,23	21,2			0,995	0,952	0,975	0,793	0,71	0,236	**0,003**
**3**	**CM-Hyp**	61,8	55,0	68,5	3,23	21,2				1,000	1,000	0,998	0,994	0,783	0,052
**4**	**CM-Hyp**	60,4	53,7	67,2	3,23	21,2					1,000	1,000	1,000	0,934	0,127
**5**	**CM-Hyp**	60,9	54,2	67,6	3,23	21,2						1,000	0,999	0,895	0,096
**6**	**CM-Hyp**	58,6	51,5	65,8	3,48	27,2							1,000	0,998	0,413
**7**	**CM-Hyp**	58,1	51,0	65,2	3,48	27,2								0,999	0,5
**8**	**CM-Hyp**	55,3	48,1	62,4	3,48	27,2									0,915
**9**	**CM-Hyp**	49,6	42,4	56,7	3,48	27,2									

Supplementary Figure S1

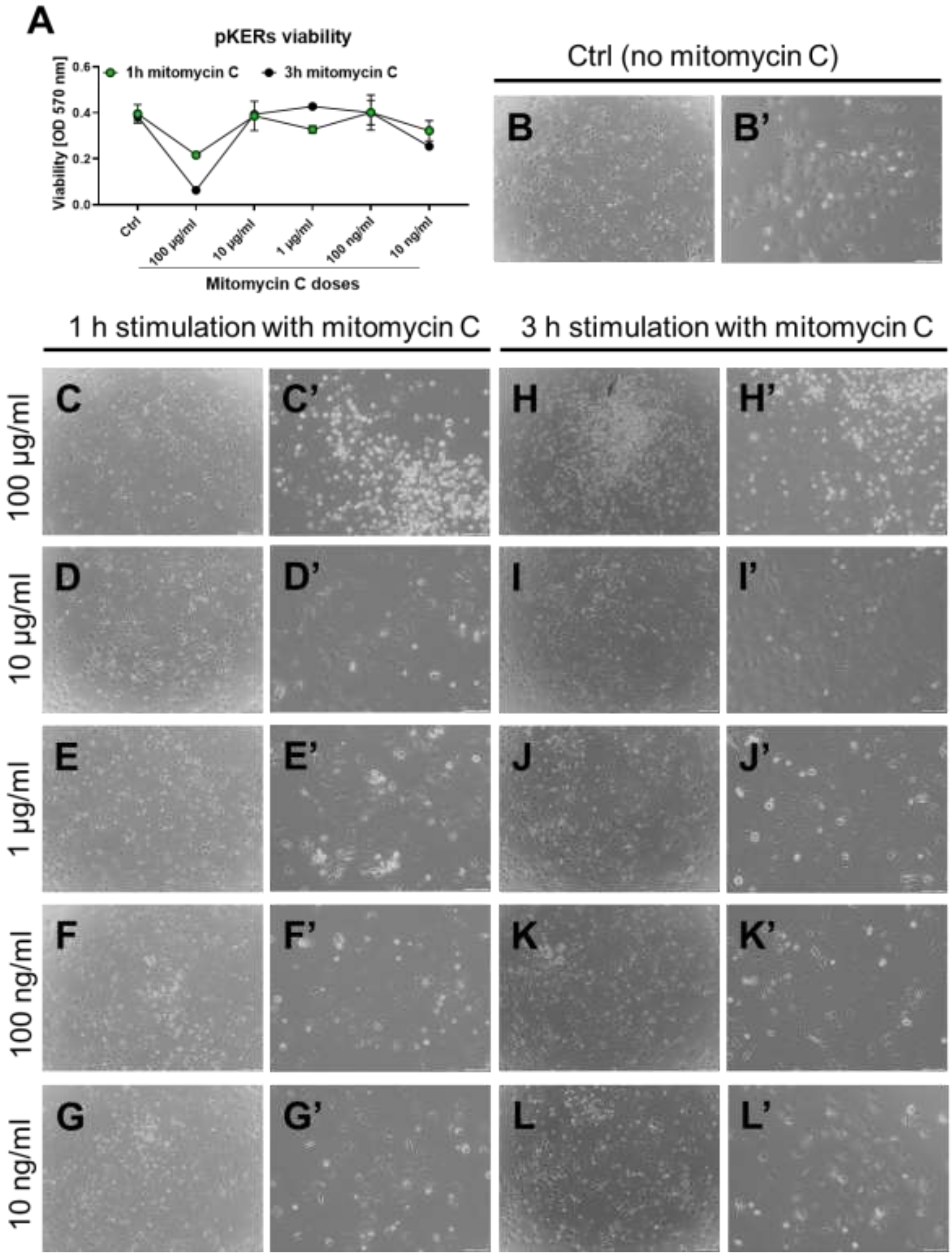

Supplementary Figure S1.
Dose and time response of pKERs (*p* = 1) to mitomycin C stimulation.

Cells were seeded onto 24-well plates at a density of 0.5 × 10^6 per well and allow to growth in keratinocyte culture medium (EpiLife™; Gibco, Life Technologies Corporation, Grand Island, NY, USA). After 48 h, the cells were treated with mitomycin C (Sigma-Aldrich Co., St. Louis, MO, USA) at the concentrations of 100 µg/mL, 10 µg/mL, 1 µg/mL, 100 ng/mL, or 10 ng/mL in keratinocyte culture medium for 3 h or 1 h, cultures with no mitomycin C were used as control. Next, the treatment was removed, cells were washed with PBS and fresh keratinocyte culture medium was added. After 24 h toxicity of mitomycin C was examined via cell viability assay based on MTT (3-[4,5-dimethylthiazol-2-yl]- 2,5-diphenyltetrazolium bromide; Sigma-Aldrich Co., St. Louis, MO, USA) colorimetric method [1].
There were no differences in cell viability between control cells (Ctrl; A, B, B′) and pKERs pre-treated with mitomycin C at doses 10 µg/ml, 1 µg/ml, 100 ng/ml, or 10 ng/ml, regardless of the exposure time (1 h or 3 h; A, D – L′). However, higher dose of mitomycicn C (100 µg/ml) showed toxic effect on pKERs manifested by the decrease in their viability and their massive detachment from the growth surface (A, C, C′, H, H′). Mitomycin C at dose of 10 µg/ml that was used in our and other studies [1-5] had no effect on pKERs viability as compared to control-treated cells (A, B, B′, D, D′, I, I′) and relative to lower doses of the treatment (A, E-L′). These results indicate that the concentration of 10 µg/ml mitomycin C is the highest effective dose that subsequently has no toxic effect on the cell viability. Moreover, no differences between both tested time points (1 h or 3 h) were observed for mitomycin C at doses 10 µg/ml, 1 µg/ml, 100 ng/ml, or 10 ng/ml (A, B, B′, D-L′). However, exposure to dose 100 µg/ml for 3 h led to greater decrease in cell viability as compared to the effect observed after 1 h of administration with the treatment (A, C-H′). Therefore, to minimize the negative effect of mitomycin C pKERs were pre-treated for 1 h. Together, these comparative data indicate that pre-tretment with mitomycin C at the concentration of 10 µg/ml for 1 h has no toxic effect on pKERs and the condition does not contribute to the cell detachment from the growth surface in wound migration assay.

Figure S1 abbreviations: pKERs; pig keratinocytes. Scale bars: 200 µm (B-L); 100 µm (B′-L′).

<u>References:</u>
[1] Gawronska-Kozak B, Kirk-Ballard H. Cyclosporin A reduces matrix metalloproteinases and collagen expression in dermal fibroblasts from regenerative FOXN1 deficient (nude) mice. Fibrogenesis Tissue Repair. 2013 Apr 2;6(1):7. doi: 10.1186/1755-1536-6-7. PMID: 23547542; PMCID: PMC3637475.
[2] Bukowska J, Kopcewicz M, Kur-Piotrowska A, Szostek-Mioduchowska AZ, Walendzik K, Gawronska-Kozak B. Effect of TGFβ1, TGFβ3 and keratinocyte conditioned media on functional characteristics of dermal fibroblasts derived from

reparative (Balb/c) and regenerative (Foxn1 deficient; nude) mouse models. Cell Tissue Res. 2018 Oct;374(1):149-163. doi: 10.1007/s00441-018-2836-8. Epub 2018 Apr 10. PMID: 29637306; PMCID: PMC6132647.
[3] Bukowska, J., Słowińska, M., Cierniak, P. *et al.* The effect of hypoxia on the proteomic signature of pig adipose-derived stromal/stem cells (pASCs). *Sci Rep* **10,** 20035 (2020). https://doi.org/10.1038/s41598-020-76796-7.
[4] Kur-Piotrowska, A., Bukowska, J., Kopcewicz, M.M. *et al.* Foxn1 expression in keratinocytes is stimulated by hypoxia: further evidence of its role in skin wound healing. *Sci Rep* **8,** 5425 (2018). https://doi.org/10.1038/s41598-018-23794-5.
[5] Kyriakides TR, Wulsin D, Skokos EA, Fleckman P, Pirrone A, Shipley JM, Senior RM, Bornstein P. Mice that lack matrix metalloproteinase-9 display delayed wound healing associated with delayed reepithelization and disordered collagen fibrillogenesis. Matrix Biol. 2009 Mar;28(2):65-73. doi: 10.1016/j.matbio.2009.01.001. Epub 2009 Jan 20. PMID: 19379668; PMCID: PMC2673333.
